# Higher risk of herpes zoster in stroke patients

**DOI:** 10.1371/journal.pone.0228409

**Published:** 2020-02-04

**Authors:** Yi- Ching Tung, Hung-Pin Tu, Ming-Kung Wu, Keng-Liang Kuo, Yu-Feng Su, Ying-Yi Lu, Chih-Lung Lin, Chieh-Hsin Wu

**Affiliations:** 1 Department of Public Health and Environmental Medicine, College of Medicine, Kaohsiung Medical University, Kaohsiung, Taiwan; 2 Department of Psychiatry, Kaohsiung Chang Gung Memorial Hospital and Chang Gung University College of Medicine, Kaohsiung, Taiwan; 3 Graduate Institute of Medicine, College of Medicine, Kaohsiung Medical University, Kaohsiung, Taiwan; 4 Division of Neurosurgery, Department of Surgery, Kaohsiung Medical University Hospital, Kaohsiung, Taiwan; 5 Department of Surgery, School of Medicine, College of Medicine, Kaohsiung Medical University, Kaohsiung, Taiwan; 6 Department of Dermatology, Kaohsiung Veterans General Hospital, Kaohsiung, Taiwan; University of Ioannina School of Medicine, GREECE

## Abstract

**Background:**

Stroke is a leading cause of death, morbidity and disability worldwide. Infection is a common complication in the acute phase after stroke. Herpes zoster is a common viral disease, in which the most debilitating complication is post-herpetic neuralgia, which can have a very large negative impact on quality of life. The aim of this study was to investigate whether stroke increases the risk of herpes zoster.

**Methods:**

This cohort study compared patients who had herpes zoster with and without a first incident of stroke. The Taiwan National Health Insurance Research Database was utilized to identify 20,551 stroke patients and 20,551 controls matched for age, gender, age categories and Charlson Comorbidity Index (CCI) score categories at a one-to-one ratio. Cox proportional-hazards regression models were employed to estimate herpes zoster risk in the stroke group relative to general population.

**Results:**

Compared to the control group, the stroke group had a greater risk for herpes zoster, especially within 1 year after stroke (adjust HR = 25.27). Both hemorrhagic stroke and ischemic stroke were significantly associated with herpes zoster (hemorrhagic type (IRR = 2.31, 95% CI, 1.67–3.20); ischemic type (IRR = 2.51, 95% CI 2.09–3.02)). However, the hemorrhagic stroke patients had a higher risk of herpes zoster ophthalmicus (IRR = 12.46, 95% CI 4.00–38.76) whereas the ischemic stroke patients had a higher risk of post-herpetic neuralgia (IRR = 2.24, 95% CI 1.56–3.20).

**Conclusion:**

Physicians should know about that adults with stroke have a higher than normal risk of herpes zoster. Thus, physicians must be acquainted with proper antiviral therapy and pain control to bring down the morbidity that ensues from herpes zoster. Use of herpes zoster vaccine may be considered in stroke patients.

## Introduction

Stroke is a leading cause of death, morbidity and disability worldwide[1]. Because stroke is a multifactorial disease resulting from interactions among many risk factors, the mechanisms of stroke differ widely. Although atherosclerosis (12–54%,) and embolism (10–26%,)[2] are the most common reasons of stroke, a substantial proportion of strokes are attributed to uncommon mechanisms namely arterial dissection (25%), vasculitis (3–5%) and coagulopathies (10%), especially among individuals aged less than 50 [3] Common risk factors include smoking, atrial fibrillation, hypertension and other cardiovascular factors. Population ageing is also increasing the incidence of stroke[4].

Herpes zoster (HZ) is caused by reactivation of a latent varicella-zoster virus (VZV) residing in sensory ganglia and dorsal roots after primary infection[5]. This disease is characterized by painful vesicular skin eruptions and related neurological disorders, which are usually unilaterally grouped and limited to one to three dermatomes. Estimates of the lifetime risk for HZ range from 10% to 30%, and the incidence and severity conspicuously increase over 50 years of age[6, 7]. Although HZ usually subsides spontaneously, 13–25% of patients undergo post-herpetic neuralgia continuing 3 months or longer[8]. Post-herpetic neuralgia, which is the most common and debilitating complication of HZ, often has a profoundly negative quality of life impact[9]. Additionally, population studies indicate that HZ increases the risks of stroke and cancer [10–12]. People suffered from HZ or HZ ophthalmicus were reported to have 1.3 to 4-fold increased risks of developing stroke, especially within one year after HZ occurrence[13]. Both ischemic and hemorrhagic strokes have been described [14]. Since the substantial health care burden imparted by HZ and its complications, patients with HZ should be educated in risk factors for stroke[15–17].

Post-stroke infection, which reportedly occurs in 23–65% of stroke patients, is associated with increased mortality and poor patient outcome [18]. Even though the reported infection rates following stroke differ enormously, infection is still a common complication during the acute post-stroke phase[19]. Infection rates in stroke patients are related to their clinical characteristics, such as stroke severity, level of consciousness, admission to ICU other than age and gender[20]. In one case-control study in Korea, Seo *et al*. showed a significant increased risk of severe HZ requiring hospitalization in ischemic stroke. However, no studies have linked the risk of HZ occurrence to hemorrhagic stroke[21]. Therefore, the aim of this study was to survey whether HZ risk is higher in persons with stroke compared to the general population and the relevance to different type of stroke. This study took advantage of the Longitudinal Health Insurance Database (LHID) to identify high-risk patients who could be focused for preventive strategies.

## Materials and methods

### Data source

The National Health Insurance (NHI) program of Taiwan is a mandatory, single-payer system established in 1995; approximately 98% of all Taiwan residents are currently enrolled, and almost all medical care providers in Taiwan are contracted to provide outpatient and inpatient services. The NHI claims records include information for inpatient, ambulatory, and home medical care. Currently, the National Health Insurance Research Database (NHIRD) is among the largest nationwide population-based databases in the world. The complete NHIRD and several dozen extracted datasets are available to researchers. Double-scrambling protocol is managed to deal with the original information so that each individual is encrypted to protect privacy. Therefore, patients can be followed up by linking claims for individual patients across the extracted datasets noted above. The LHID2010 constitutes registration and claims data collected by the NHI program for a nationally representative group of 1 million individuals. All participants were enrolled in the NHI during the period from January 1, 1996, to December 31, 2010. The design of this study was reviewed and approved by the Institutional Review Board of Chang Gung Medical Foundation (Number 201900594B1).

### Study population and potential confounders

The HZ risk after stroke was estimated by analysis of a nationwide population-based cohort. The cohort dataset used in the analysis included patients with and without a first-time incident of stroke and HZ. The study cohort included patients older than 18 years who were still alive in 2010. All diagnoses of patients with linked data were coded according to the International Classification of Diseases, Ninth Revision, Clinical Modification (ICD-9-CM). The dataset for this cohort study included 20,551 stroke cases that required hospitalization since January 1, 1996 (hemorrhagic stroke, ICD-9-CM = 430–432; ischemic stroke, ICD-9-CM = 433–434; undefined stroke, ICD-9-CM = 435–438). Propensity score matching method was used to match the patients with stroke to a control group of 20,551 subjects without stroke for age, gender, age categories and Charlson Comorbidity Index (CCI) score categories. The CCI scores were calculated as follows: myocardial infarction (1 point), congestive heart failure (1 point), peripheral vascular disease (1 point), dementia (1 point), chronic pulmonary disease (1 point), rheumatologic disease (1 point), peptic ulcer disease (1 point), mild liver disease (1 point), diabetes mellitus (1 point uncomplicated), diabetes mellitus (2 points if end-organ damage), hemiplegia or paraplegia (2 points), renal disease (1 point), any malignancy (2 points), liver disease (3 points if moderate to severe), metastatic solid tumor (6 points), and AIDS (6 points)[22]. The CCI scores were divided into four categories (score = 0, 1–2, 3–4, and ≥5). Stroke was excluded from the calculations of CCI score.

### Clinical outcomes

Herpes zoster was defined according to ICD-9-CM coding system as HZ with ophthalmicus (053.2), other trigeminal area (053.12), otitis externa (053.71), or unspecified site (053.x)requiring hospitalization or outpatient treatment. Post-herpetic neuralgia was defined as 053.1. To limit the analysis to patients with a first-time incident of stroke or HZ, individuals were excluded if they had any evidence of stroke or HZ before the study period (n = 1557). The subjects were followed up for development of HZ during the next 15 years (1996–2010).

### Self-controlled case-series study (SCCS) method (Fig 1)

**Fig 1:**
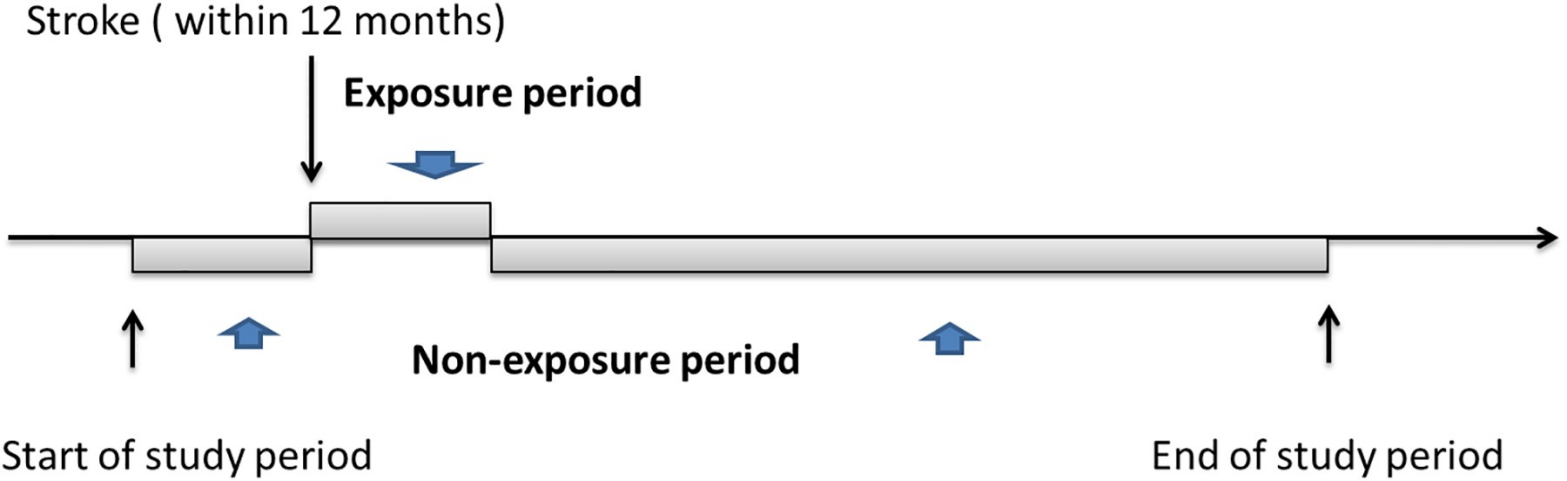
Pictorial representation of the self-controlled case series study.

The SCCS method was used to compare within-patient risks during different time periods. All patients who had both a stroke accident and HZ event were identified. Since it’s hard to collect data for the confounding factors completely, observational study is not able to effectively capture crucial differences between patients with and without stroke, which leads to residual confounding[23]. As a result, risks are usually compared between the post-stroke period and other “non-exposure” time periods. Avoiding between-person confounding effects resulted from different HZ risks between individuals without or with stroke is the major advantage through utilizing SCCS.

### Statistical analysis

Parametric continuous data were compared across groups by Student t-test,one-way analysis of variance or Kruskal-Wallis test when categorical data were compared by Chi-square test as appropriate. Survival times were determined across from stroke occurrence to the date of theHZ event or to the end of the study period (December 31, 2010), whichever occurred first. Kaplan-Meier method was used to construct the survival curves, and log-rank test was used to compare homogeneity between groups. The hazard ratios (HRs) and 95% confidence intervals (CIs) for HZ events were estimated by Cox proportional hazards model after adjustment for age, gender and CCI. Poisson regression analysis was performed to calculate incident rate ratio (IRR).

The SCCS method was used for within-patient comparisons of risks during different time periods. The exposure period began with the onset of stroke accident and ended 12 months later. The non-exposure time period was defined as all other observation time period. The primary analyses estimated the effects of different stroke types (hemorrhagic, ischemic, and undefined) on the risk of HZ; further analyses were done to assess the different types of HZ risk such as ophthalmic, other trigeminal, unspecified-site, and post-herpetic neuralgia. Conditional Poisson regression analysis was utilized to determine IRRs and 95% CIs for HZ within each stratum of the exposure period in comparison with non-exposure time period after adjustment for age at the index date for HZ and gender.

All data were processed and analyzed using Statistical Analysis Software, version 9.4 (SAS Institute, Cary, NC, USA) with a statistically significant level of 2-tailed P-value < 0.05

## Results

### Characteristics of the study population

Table 1 presents the characteristics of the study population. Average age in the 20,551 stroke patients and 20,551 matched controls was 68.8±13.5 and 68.7±13.3 years, respectively. The stroke patients and controls did not significantly differ in age groups, gender, or CCI scores.

**Table 1:** Characteristics of subjects with stroke and matched controls.

	Stroke		
Variables	Yes = 20,551	No = 20,551	P-value
Age mean(SD), years	68.8 (13.5)	68.7 (13.3)	0.5153
Median (IQR), years	70.6 (59.7–79.1)	70.3 (59.8–78.9)	0.2141
Age group, n(%)			
18–29	181(0.9)	145(0.7)	
30–39	417(2.0)	387(1.9)	
40–49	1234(6.0)	1347(6.6)	
50–59	3475(16.9)	3364(16.4)	
60–69	4644(22.6)	4777(23.2)	
70–79	6069(29.5)	6109(29.7)	
≥80	4531(22.1)	4422(21.5)	0.8267
Gender, n(%)			
Males	11417(55.6)	11463(55.8)	
Females	9134(44.5)	9088(44.2)	0.6479
Charlson Comorbidity Index,n(%)			
0	1281(6.2)	1291(6.3)	
1–2	4832(23.5)	4621(22.5)	
3–4	5849(28.5)	6147(29.9)	
≥5	8589(41.8)	8492(41.3)	0.6235

SD: standard deviation; IQR: interquartile range.

### Stroke and HZ risk (Table 2)

**Table 2:** Stroke associated with herpes zoster stratified by gender, age and by 1-year and 5-year follow-up period.

Variables	Herpes zoster	Person-years at risk	Rate	IRR (95% CI)	P	Adjusted HR* (95% CI)	P
1 year following stroke							
Male cohort set							
Controls	8	11459.06	0.70	1.00		1.00	
Stroke cases	145	10743.49	13.50	19.33 (9.48–39.39)	<0.0001	19.74 (9.7–40.21)	<0.0001
Female cohort set							
Controls	3	9087.01	0.33	1.00		1.00	
Stroke cases	117	8650.38	13.53	40.97 (13.02–128.87)	<0.0001	40.44 (12.86–127.23)	<0.0001
Age group <65 years							
Controls	1	7449.48	0.13	1.00		1.00	
Stroke cases	80	7019.78	11.40	84.90 (11.81–610.09)	<0.0001	86.04 (11.97–618.4)	<0.0001
Age group ≥65 years							
Controls	10	13096.59	0.76	1.00		1.00	
Stroke cases	182	12374.09	14.71	19.26 (10.19–36.41)	<0.0001	19.40 (10.27–36.66)	<0.0001
Total cohort set							
Controls	11	20546.07	0.54	1.00		1.00	
Stroke cases	262	19393.87	13.51	25.23 (13.80–46.13)	<0.0001	25.27 (13.82–46.19)	<0.0001
5 yearsfollowing stroke							
Male cohort set							
Controls	218	57021.21	3.82	1.00		1.00	
Stroke cases	477	41864.83	11.39	2.98 (2.54–3.50)	<0.0001	3.16 (2.69–3.71)	<0.0001
Female cohort set							
Controls	153	45251.98	3.38	1.00		1.00	
Stroke cases	453	34497.05	13.13	3.88 (3.23–4.67)	<0.0001	3.91 (3.25–4.70)	<0.0001
Age group <65 years							
Controls	68	37168.93	1.83	1.00		1.00	
Stroke cases	257	26934.49	9.54	5.22 (3.99–6.81)	<0.0001	5.38 (4.11–7.04)	<0.0001
Age group ≥65 years							
Controls	303	65104.26	4.65	1.00		1.00	
Stroke cases	673	49427.39	13.62	2.93 (2.55–3.35)	<0.0001	3.03 (2.64–3.47)	<0.0001
Total cohort set							
Controls	371	102273.19	3.63	1.00		1.00	
Stroke cases	930	76361.88	12.18	3.36 (2.98–3.79)	<0.0001	3.44 (3.05–3.88)	<0.0001

95%CI = 95% confidence interval, Rate = incidence per 1000 person-years, IRR = incident rate ratio, HR = hazard ratio.

* Model adjusted for age, CCI score and, for the combined group, gender, using a Cox proportional-hazards regression model.

In this cohort set, 1,539 of 20,551 stroke cases and 2,844 of 20,551 matched controls experienced HZ events. Interestingly, at the 1-year follow up and 5-year follow up, 262 and 930 of 20,551 stroke patients had HZ whereas only 11 and 371 of 20,551 matched controls had HZ. That is, the incidence of HZ over a 1-year period was significantly greater in stroke patients (13.51 per 1000 person-years) compared to controls (0.54 per 1000 person-years). Furthermore, the incidence of HZ over a 5-year period was also significantly greater in stroke patients (12.18per 1000 person-years) compared to controls (3.63 per 1000 person-years). Our results showed that stroke significantly (P<0.0001) increased the risk of HZ events over a 1-year period (adjusted HR = 25.27) and over a 5-year period (adjusted HR = 3.44). Stratification by gender and age similarly showed a significantly higher risk in stroke patients compared to controls. In stroke patients, the incidence of HZ did not significantly differ between males and females, but increase with age.

**Fig 2:**
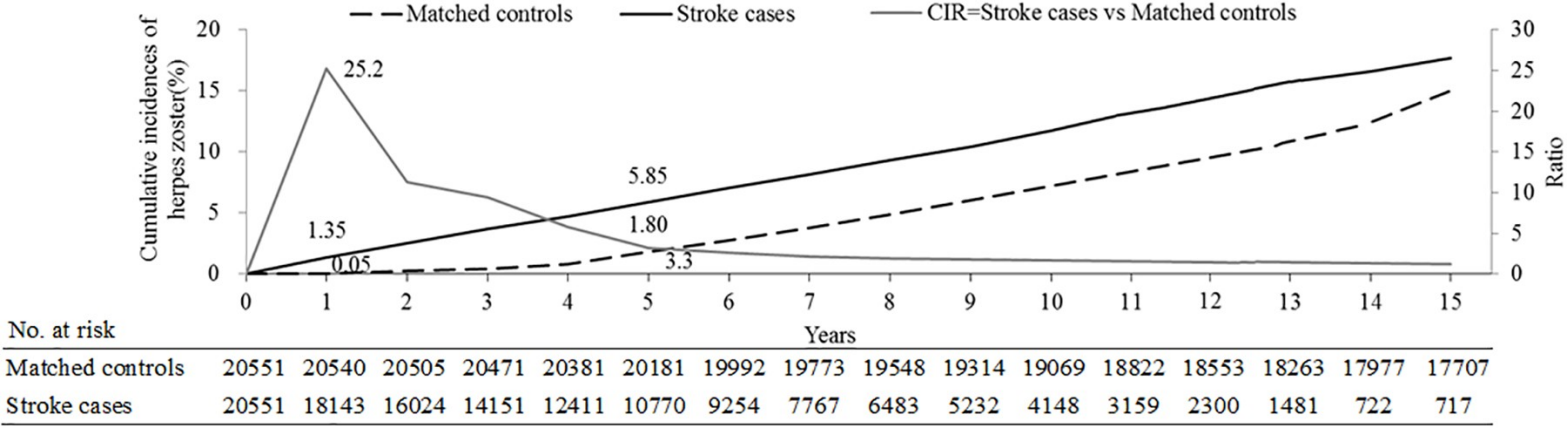
Cumulative incidences of herpes zoster.

Fig 2 shows that the Kaplan–Meier curves for incidence of HZ events significantly differed between individuals with and without stroke (log-rank test P< 0.0001). In patients with stroke, the cumulative incidence of HZ was 1.35% over a 1-year period (versus 0.05% in controls) and 5.85% over a 5-year period (versus 1.80% in controls).

Next, a self-controlled case-series study method was used to compare within-patient (n = 1,539) stroke risk during different time periods.

Table 3 shows that the median age at time of stroke was 65.1 years (interquartile range [IQR] = 55.9–72.5 years), and 51.1% of stroke patients were male. The median observation period was 3.8 years (IQR = 1.6–6.9 years). Most strokes were ischemic type (n = 777), followed by undefined type (n = 512) and hemorrhagic type (n = 250). Most patients (n = 1,424) suffered from the HZ of unspecified-site; 83 patients had HZ ophthalmicus; 32 patients had HZ in other branches of the trigeminal nerve; 417 patients had post-herpetic neuralgia.

**Table 3:** Characteristics of stroke patients with herpes zoster.

Variables	Overall stroke	Stroke type			P value
		Hemorrhagic	Ischemic	Undefined	
N	1539	250	777	512	
Age at index herpes zoster, years, mean(SD)	64.0(11.7)	60.4(13.3)	65.4(10.9)	63.6(11.7)	<0.0001
Median (IQR)	65.1 (55.9–72.5)	60.6 (51.3–70.5)	66.4 (57.9–73.1)	64.4 (55.3–72.0)	<0.0001
Males, n (%)	786 (51.1)	144 (57.6)	395 (50.8)	247 (48.2)	0.0518
Total observation, years, median (IQR)	3.8 (1.6–6.9)	3.8 (1.3–6.9)	3.6 (1.5–6.5)	4.1 (1.8–7.3)	0.0650
Type of herpes zoster, n(%)					
Ophthalmic	83 (5.4)	12 (4.8)	53 (6.8)	18 (3.5)	0.0331
Other trigeminal	32 (2.1)	4 (1.6)	23 (3.00)	5 (1.00)	0.0428
Unspecified Site	1424 (92.5)	234(93.6)	701(90.2)	489(95.5)	0.0015
Post-herpetic neuralgia	417(27.1)	58(23.2)	224(28.8)	135(26.4)	0.1979

SD: standard deviation; IQR: interquartile range.

Table 4 shows that, compared to non-exposure period, the incidence rates of HZ and post-herpetic neuralgia significantly increased. (Overall HZ cases (IRR = 2.23, 95% CI, 1.95–2.55), patients with HZ ophthalmicus (IRR = 2.46, 95% CI 1.40–4.33), patients with unspecified-site HZ (IRR = 2.23, 95% CI 1.94–2.56) and patients with post-herpetic neuralgia (IRR = 2.15, 95% CI 1.65–2.81)). Our results also showed that HZ risk was increased by each stroke type, including hemorrhagic type (IRR = 2.31, 95% CI, 1.67–3.20); ischemic type (IRR = 2.51, 95% CI 2.09–3.02); and undefined type (IRR = 1.80, 95% CI 1.40–2.30). Analysis by stroke type further revealed risk of HZ ophthalmicus was highest in patients with hemorrhagicstroke (IRR = 12.46, 95% CI 4.00–38.76) and that risk of post-herpetic neuralgia was highest in patients with ischemic stroke (IRR = 2.24, 95% CI 1.56–3.20).

**Table 4:** Adjusted incidence rate ratios of herpes zoster during risk periods of 12 months following stroke.

Variables	Overall herpes zoster		Herpes zoster type						Post-herpetic neuralgia	
			Ophthalmic		Other trigeminal		Unspecified Site			
	IRR* (95% CI)	P	IRR* (95% CI)	P	IRR* (95% CI)	P	IRR * (95% CI)	P	IRR* (95% CI)	P
Overall stroke	2.23 (1.95–2.55)	<0.0001	2.46 (1.40–4.33)	<0.0001	1.63 (0.57–4.66)	0.3636	2.23 (1.94–2.56)	<0.0001	2.15 (1.65–2.81)	<0.0001
Stroke types										
Hemorrhagic	2.31 (1.67–3.20)	<0.0001	**12.46 (4.00–38.76)**	**<0.0001**	4.59 (0.48–44.17)	0.1869	2.01 (1.42–2.85)	<0.0001	1.63 (0.77–3.45)	0.2049
Ischemic	2.51 (2.09–3.02)	<0.0001	1.43 (0.61–3.35)	0.4126	0.48 (0.06–3.60)	0.4767	2.69 (2.22–3.25)	<0.0001	**2.24 (1.56–3.20)**	<0.0001
Undefined	1.80 (1.40–2.30)	<0.0001	2.05 (0.58–7.19)	0.2633	9.08 (1.51–54.44)	0.0158	1.75 (1.35–2.26)	<0.0001	2.27 (1.43–3.59)	0.0005

95% CI = 95% confidence interval, IRR = incident rate ratio.

*Model adjusted for age and gender using a conditional Poisson regression model.

## Discussion

After controlling for covariates, patients with stroke had a 25.27 times higher risk of HZ compared to the general population over a 1-year follow-up period and a 3.44 times higher risk of HZ over a 5-year follow-up period. After stratification by gender, the incidence of HZ events was significantly higher in both male and female stroke patients compared to controls. Furthermore, the incidence of HZ events was significantly higher in stroke patients at different ages compared to controls after the stratification by age. Both hemorrhagic stroke and ischemic stroke were significantly associated with increased risk of HZ. Specifically, hemorrhagic stroke was associated with an increased risk of HZ ophthalmicus and ischemic strokewas associated with an increased risk of post-herpetic neuralgia. To our knowledge, this is the first cohort study to compare HZ risk between hemorrhagic stroke and ischemic stroke.

Risk factors for HZ include female gender, mechanical trauma, genetic susceptibility,[24] interleukin 10 gene polymorphism[25] as well as weakening of the immune system due to the aging process[26], comorbidity[27], drug exposure[28], disease severity[29] and nutritional deficiencies[15]. The causal pathway between stroke and HZ remains unclear but is probably multifactorial.

Firstly, stroke results in a localized depletion of oxygen and energy, which causes death of cells in affected areas[30]. Dying cells stimulate an inflammatory response intervened by local innate immune cells in the brain and other immune cells entering into the brain from the bloodstream. Inflammatory reactions release inhibitory molecules within the immune system, which are usually self-limiting and self-resolving. The release of pro-inflammatory cytokines in the brain stimulates the expansion of an immune-suppressive cell population which suppresses both innate and adaptive immune responses^4^. The excessive inflammatory response in the brain paradoxically prompts an immune-suppression state in the peripheral tissues. Therefore, stroke patients are at risk for fatal secondary infections[31, 32]. On one hand, the autonomic nervous system controls inflammation through neural circuits that affect immune cells[33]. Cholinergic anti-inflammatory pathways sense inflammation through peripheral nerves and then suppress pro-inflammatory cytokines production[34]. On the other hand, myeloid-derived suppressor cells (MDSCs) present adaptive immune responses and release anti-inflammatory factors which interrupt the destructive cycle of chronic inflammation[35]. Activities of MDSCs are not limited to the central nervous system but also occur in the periphery tissues. Lies *et al*. reported that MDSCscirculating in the blood stream can shape systemic inflammatory responses that may induce post-stroke immune-suppression[31]. Patients who develop post-stroke infections tend to have a reduced lymphocyte count and a delayed recovery of T-lymphocyte loss. Suppression of the immune system inducing post-stroke infection can then increase the risk of HZ infection.

Secondly, depression is a common complication after stroke[36]; post-stroke depression affects up to one third of stroke survivors[37]. The depression risk in stroke survivors almost doubles that in the general population. As many as half of all stroke survivors experience depression within 5 years after stroke[38]. Depression risk may be affected by the extent and location of brain injury, vascular comorbidities, and reaction to a new functional disability[39]. Studies consistently show that physical disability, cognitive impairment and high stroke severity are risk factors for depression[40]. Persistent depression at 12 months after stroke is related to female gender, young age, non-White ethnicity, inability to work and poor functional outcome. Depression is known to have neuro-endocrineand immunological/ inflammationeffects[41,42]. Sustainedactivationof the hypothalamic-pituitary-adrenal axis and the sympathetic nervous system during chronic stress leads to immunosuppression in mood disorders[43]. Mood disorders including anxiety or depression are linked to impaired cell mediated immunity (CMI)[43]. Herpes zoster results from reactivation of the VZV from sensory ganglia while cell-mediated immunity declines below a critical level[44]. Specifically, CMI specific to VZV is decreased in major depression and is negatively related to severity[45, 46]. In two case-control studies using data from practices in the United Kingdom and nationwide Danish registries[47], Schmidt *et al*. showed that mood disorders, including depression, were associated with increased risk of HZ. In a retrospective population-based cohort study, Liao *et al*. revealed that patients with depression are at a raised risk of HZ, especially those aged 45 to 54 years and those with comorbidities, including hypertension, hyperlipidemia, renal diseases, rheumatic diseases, anxiety, sleep disorder and malignant conditions[48]. Depression is also associated with obesity, smoking and low physical activity[49–51]. Furthermore, depression increases mortality and worsens preexisting morbidities including diabetes and cardiovascular disease [52–54], both of which are risk factors for HZ[55]. Hence, we hypothesize that reduced cell-mediated immunity caused by post-stroke depression may be a risk factor for the development of HZ, especially during the time period shortly after stroke.

Herpes zoster is mostly observed in elderly immunocompromisedpatients, which is consistent with our result which showed the incidence of HZ increased with age in stroke patients[56]. Literatures reported that many infections occurred during acute stroke phase, which developed 3 days after stroke event[20]. In the current study,the Cox proportional hazards model and the Kaplan-Meier analysis demonstrated that the risk of herpes zosterdeveloped 1 year after stroke and continued over time, even after 5 years of stroke event. These findings stress that the immunological dysfunctions after stroke may be rapid onset and last for a long time; hence, preventive strategies for herpes zoster infection following strokeshould not be interrupted. Both the risks for hemorrhagic stroke and ischemic stroke increased after a HZ episode. The risk of developing HZ ophthalmicus is highest in hemorrhagic strokewhile post-herpetic neuralgia is highest in ischemic stroke. Older age is the most important attributable factor in stroke and HZ. Although the presence of immunosuppression could exacerbate the HZ ophthalmicus and post-herpetic neuralgia, the mechanism of stroke subtypes influence attack of HZ ophthalmicus or post-herpetic neuralgia remains unknown. Furtherexploration into this matter is considered.

A notable strength of this study is its use of a large-population database, which provided sufficient statistical power to analyses. Secondly, this study used propensity-score matching to lessen confounded associations and selection bias. Another strength is its analysis of a relatively homogenous Taiwan population. Most subjects were ethnically Han Chinese, which reduces potential confounding effects of racial differences since ethnicity may be a risk factor for HZ[57].

However, several limitations should be considered in this analysis. First, misclassification is possible because the HZ diagnoses were based on ICD-9 codes by physicians. However, the clinical presentations of HZ are characteristic, and patients in Taiwan do not require a referral letter from a general practitioner before their initial visit with a dermatologist. Hence, an HZ misdiagnosis was probably a rare occurrence in our study population. Although some patients with mild HZ may not seek medical help, the number of patients with HZ who did not visit a physician was probably low because the Taiwan health insurance system requires only a small copayment for each visit. Second, the database lacks information regarding possible confounding factors such as smoking habits, alcohol consumption, dietary habits, nutritional status, physical activities and laboratory confirmations. Additionally, the administrative database did not contain information about stroke severity or location and level of consciousness; hence, the effects of these factors on the occurrence of HZ could not be determined. Furthermore, it was not possible to evaluate all risk factors and to avoid all confounders. Most comorbidities related to the risk of HZ were identified in CCI except depression. However, propensity-score matching was utilized in our analysis; hence, all adjusted HRs for HZ in all subclasses of stroke patients indicated increased HZ risk, which validates the findings of this study. Moreover, recurrent stroke among those stroke patients experiencing HZ episode can’t be easily identified just by using ICD-9-CM codes. Finally, further research is warranted although the mechanisms of post-stroke HZ infections arebeyond the scope of the current study.

## Conclusion

In conclusion, this population-based study showed that, in comparison with a control group without stroke, adult stroke patients have an increased risk for HZ, especially within 1 year after stroke. Both hemorrhagic stroke and ischemic stroke were significantly associated with HZ risk. Moreover, hemorrhagic stroke had a strong association with HZ ophthalmicus, and ischemic stroke had a strong association with post-herpetic neuralgia. Post-herpetic neuralgia lasting for months or years is the major morbidity caused by HZ, which consumes substantial medical resources[7]; treating the complication often requires a multifaceted approach. Herpes zoster vaccines are now considered effective at markedly reducing morbidity related to HZ in the immunocompetent elderly[22]. Physician should know about the increased incidence of HZ in adults with stroke and should be acquainted with proper antiviral therapy and pain control to reduce the morbidity that ensues from an HZ outbreak.

## References

[1] Goldstein LB, Bushnell CD, Adams RJ, Appel LJ, Braun LT, Chaturvedi S, et al Guidelines for the primary prevention of stroke: a guideline for healthcareprofessionals from the American Heart Association/American Stroke Association.Stroke. 2011;42(2):517–84. 10.1161/STR.0b013e3181fcb238 21127304

[2] Hankey GJ. Stroke. Lancet. 2017;389(10069):641–54 10.1016/S0140-6736(16)30962-X 27637676

[3] Alexander B, Philipp G, Peter DS. Rare specific causes of stroke In: Stefan S, Daniel H, editors. Critical Care of the Stroke Patient. Cambridge: Cambridge University Press;2014 pp 226–28

[4] Mackay J, Mensah GA, Mendis S, Greenlund K, World Health Organization. The atlas of heart disease and stroke. Geneva: World Health Organization; 2004 112

[5] Jih JS, Chen YJ, Lin MW, Chen YC, Chen TJ, Huang YL, et al Epidemiological features and costs of herpes zoster in Taiwan: a national study 2000 to 2006. Acta Derm Venereol. 2009;89(6):612–6. 10.2340/00015555-0729 19997693

[6] Wareham DW, Breuer J. Herpes zoster. BMJ. 2007;334(7605):1211–5. 10.1136/bmj.39206.571042.AE 17556477 PMC1889999

[7] Lin YH, Huang LM, Chang IS, Tsai FY, Lu CY, Shao PL, et al Disease burden and epidemiology of herpes zoster in pre-vaccine Taiwan. Vaccine. 2010;28(5):1217–20. 10.1016/j.vaccine.2009.11.029 19944790

[8] Yawn BP, Saddier P, Wollan PC, St Sauver JL, Kurland MJ, Sy LS. A population-based study of the incidence and complication rates of herpes zoster before zoster vaccine introduction. Mayo Clin Proc. 2007;82(11):1341–9. 10.4065/82.11.1341 17976353

[9] Yawn BP, Itzler RF, Wollan PC, Pellissier JM, Sy LS, Saddier P. Health care utilization and cost burden of herpes zoster in a community population. Mayo Clin Proc. 2009;84(9):787–94 19720776 10.4065/84.9.787 PMC2735428

[10] Lin HC, Chien CW, Ho JD. Herpes zoster ophthalmicus and the risk of stroke: a population-based follow-up study. Neurology. 2010;74(10):792–7. 10.1212/WNL.0b013e3181d31e5c 20200348

[11] Kang JH, Ho JD, Chen YH, Lin HC. Increased risk of stroke after a herpes zoster attack: a population-based follow-up study. Stroke. 2009;40(11):3443–8. 10.1161/STROKEAHA.109.562017 19815828

[12] Chiu HF, Chen BK, Yang CY. Herpes zoster and subsequent risk of cancer: a population-based study. J Epidemiol. 2013;23(3):205–10. 10.2188/jea.JE20120155 23545577 PMC3700258

[13] Wu PH, Chuang YS, Lin YT. Does Herpes Zoster Increase the Risk of Stroke and Myocardial Infarction? A Comprehensive Review. J Clin Med. 2019;8(4):547. 10.3390/jcm8040547 PMC6518274 31013629

[14] Gilden D, Cohrs RJ, Mahalingam R, Nagel MA. Varicella zoster virus vasculopathies: diverse clinical manifestations, laboratory features, pathogenesis, and treatment. Lancet Neurol. 2009;8(8):731–40. 10.1016/S1474-4422(09)70134-6 19608099 PMC2814602

[15] Thomas SL, Wheeler JG, Hall AJ. Micronutrient intake and the risk of herpes zoster: a case-control study. Int J Epidemiol. 2006;35(2):307–14. 10.1093/ije/dyi270 16330478

[16] Chen HH, Chen YM, Chen TJ, Lan JL, Lin CH, Chen DY. Risk of herpes zoster in patients with systemic lupus erythematosus: a three-year follow-up study using a nationwide population-based cohort. Clinics (Sao Paulo). 2011;66(7):1177–82. 21876970 10.1590/S1807-59322011000700009 PMC3148460

[17] Veetil BM, Myasoedova E, Matteson EL, Gabriel SE, Green AB, Crowson CS. Incidence and time trends of herpes zoster in rheumatoid arthritis: a population-based cohort study. Arthritis Care Res (Hoboken). 2013;65(6):854–61. 23281295 10.1002/acr.21928 PMC3674119

[18] Neumann S, Shields NJ, Balle T, Chebib M, Clarkson AN. Innate Immunity and Inflammation Post-Stroke: An alpha7-Nicotinic Agonist Perspective. Int J Mol Sci. 2015;16(12):29029–46. 10.3390/ijms161226141 26690125 PMC4691088

[19] Vermeij FH, Scholte op Reimer WJ, de Man P, van Oostenbrugge RJ, Franke CL, de Jong G, et al Stroke-associated infection is an independent risk factor for poor outcome after acute ischemic stroke: data from the Netherlands Stroke Survey. Cerebrovasc Dis. 2009;27(5):465–71. 10.1159/000210093 19329851

[20] Westendorp WF, Nederkoorn PJ, Vermeij JD, Dijkgraaf MG, van de Beek D. Post-stroke infection: a systematic review and meta-analysis. BMC Neurol. 2011;11:110 10.1186/1471-2377-11-110 21933425 PMC3185266

[21] Seo HM, Cha MJ, Han JH, Han K, Park SH, Bang CH, et al Reciprocal relationship between herpes zoster and cardiovascular diseases: A nationwide population-based case-control study in Korea. J Dermatol 10.1111/1346-8138.14597 30118146

[22] Quan H, Sundararajan V, Halfon P, Fong A, Burnand B, Luthi JC, et al Coding algorithms for defining comorbidities in ICD-9-CM and ICD-10 administrative data. Med Care. 2005;43(11):1130–9. 10.1097/01.mlr.0000182534.19832.83 16224307

[23] Fewell Z, Davey Smith G, Sterne JA. The impact of residual and unmeasured confounding in epidemiologic studies: a simulation study. Am J Epidemiol 2007;166(6):646–55. 10.1093/aje/kwm165 17615092

[24] Thomas SL, Hall AJ. What does epidemiology tell us about risk factors for herpes zoster? Lancet Infect Dis. 2004;4(1):26–33. 10.1016/s1473-3099(03)00857-0 14720565

[25] Zerboni L, Sen N, Oliver SL, Arvin AM. Molecular mechanisms of varicella zoster virus pathogenesis. Nat Rev Microbiol. 2014;12(3):197–210. 10.1038/nrmicro3215 24509782 PMC4066823

[26] Oxman MN, Levin MJ, Johnson GR, Schmader KE, Straus SE, Gelb LD, et al A vaccine to prevent herpes zoster and postherpetic neuralgia in older adults. N Engl J Med. 2005;352(22):2271–84. 10.1056/NEJMoa051016 15930418

[27] Joesoef RM, Harpaz R, Leung J, Bialek SR. Chronic medical conditions as risk factors for herpes zoster. Mayo Clin Proc. 2012;87(10):961–7. 10.1016/j.mayocp.2012.05.021 23036671 PMC3538398

[28] Smitten AL, Choi HK, Hochberg MC, Suissa S, Simon TA, Testa MA, et al The risk of herpes zoster in patients with rheumatoid arthritis in the United States and the United Kingdom. Arthritis Rheum. 2007;57(8):1431–8. 10.1002/art.23112 18050184

[29] Zisman D, Bitterman H, Shalom G, Feldhamer I, Comanesther D, Batat E, et al Psoriatic arthritis treatment and the risk of herpes zoster. Ann Rheum Dis. 2016;75(1):131–5. 10.1136/annrheumdis-2013-205148 25261573

[30] Dirnagl U, Iadecola C, Moskowitz MA. Pathobiology of ischaemic stroke: an integrated view. Trends Neurosci. 1999;22(9):391–7. 10.1016/s0166-2236(99)01401-0 10441299

[31] Liesz A, Dalpke A, Mracsko E, Antoine DJ, Roth S, Zhou W, et al DAMP signaling is a key pathway inducing immune modulation after brain injury. J Neurosci. 2015;35(2):583–98. 10.1523/JNEUROSCI.2439-14.2015 25589753 PMC4293412

[32] Chamorro A, Urra X, Planas AM. Infection after acute ischemic stroke: a manifestation of brain-induced immunodepression. Stroke. 2007;38(3):1097–103. 10.1161/01.STR.0000258346.68966.9d 17255542

[33] Tracey KJ. Reflex control of immunity. Nat Rev Immunol. 2009;9(6):418–28. 10.1038/nri2566 19461672 PMC4535331

[34] Cai PY, Bodhit A, Derequito R, Ansari S, Abukhalil F, Thenkabail S, et al Vagus nerve stimulation in ischemic stroke: old wine in a new bottle. Front Neurol. 2014;5:107 10.3389/fneur.2014.00107 25009531 PMC4067569

[35] Ostrand-Rosenberg S, Sinha P, Beury DW, Clements VK. Cross-talk between myeloid-derived suppressor cells (MDSC), macrophages, and dendritic cells enhances tumor-induced immune suppression. Semin Cancer Biol. 2012;22(4):275–81. 10.1016/j.semcancer.2012.01.011 22313874 PMC3701942

[36] Lewin-RichterA, VolzM, JobgesM, WerheidK. Predictivity of Early Depressive Symptoms for Post-Stroke Depression. J Nutr Health Aging. 2015;19(7):754–8. 10.1007/s12603-015-0540-x 26193859

[37] Creutzfeldt CJ, Holloway RG, Walker M. Symptomatic and palliative care for stroke survivors. J Gen Intern Med. 2012;27(7):853–60. 10.1007/s11606-011-1966-4 22258916 PMC3378740

[38] Ayerbe L, Ayis S, Rudd AG, Heuschmann PU, Wolfe CD. Natural history, predictors, and associations of depression 5 years after stroke: the South London Stroke Register. Stroke. 2011;42(7):1907–11. 10.1161/STROKEAHA.110.605808 21566241

[39] El Husseini N, Goldstein LB, Peterson ED, Zhao X, Pan W, Olson DM, et al Depression and antidepressant use after stroke and transient ischemic attack. Stroke. 2012;43(6):1609–16. 10.1161/STROKEAHA.111.643130 22461330

[40] Hackett ML, Anderson CS. Predictors of depression after stroke: a systematic review of observational studies. Stroke. 2005;36(10):2296–301. 10.1161/01.STR.0000183622.75135.a4 16179565

[41] Shimbo D, Chaplin W, Crossman D, Haas D, Davidson KW. Role of depression and inflammation in incident coronary heart disease events. Am J Cardiol. 2005;96(7):1016–21. 10.1016/j.amjcard.2005.05.064 16188535

[42] Musselman DL, Evans DL, Nemeroff CB. The relationship of depression to cardiovascular disease: epidemiology, biology, and treatment. Arch Gen Psychiatry. 1998;55(7):580–92. 10.1001/archpsyc.55.7.580 9672048

[43] Zorrilla EP, Luborsky L, McKay JR, Rosenthal R, Houldin A, Tax A, et al The relationship of depression and stressors to immunological assays: a meta-analytic review. Brain Behav Immun. 2001;15(3):199–226. 10.1006/brbi.2000.0597 11566046

[44] Wilson JF. In the clinic. Herpes zoster. Ann Intern Med. 2011;154(5):ITC31–15; quiz ITC316. 10.7326/0003-4819-154-5-201103010-01003 21357905

[45] Irwin M, Costlow C, Williams H, Artin KH, Chan CY, Stinson DL, et al Cellular immunity to varicella-zoster virus in patients with major depression. J Infect Dis. 1998;178 Suppl 1:S104–8. 9852986 10.1086/514272

[46] Irwin MR, Levin MJ, Laudenslager ML, Olmstead R, Lucko A, Lang N, et al Varicella zoster virus-specific immune responses to a herpes zoster vaccine in elderly recipients with major depression and the impact of antidepressant medications. Clin Infect Dis. 2013;56(8):1085–93. 10.1093/cid/cis1208 23413415 PMC3601721

[47] Schmidt SAJ, Langan SM, Pedersen HS, Schonheyder HC, Thomas SL, Smeeth L, et al Mood Disorders and Risk of Herpes Zoster in 2 Population-Based Case-Control Studies in Denmark and the United Kingdom. Am J Epidemiol. 2018;187(5):1019–28. 10.1093/aje/kwx338 29053820 PMC5968637

[48] Liao CH, Chang CS, Muo CH, Kao CH. High prevalence of herpes zoster in patients with depression. J Clin Psychiatry. 2015;76(9):e1099–104. 10.4088/JCP.14m09311 26455673

[49] Camacho TC, Roberts RE, Lazarus NB, Kaplan GA, Cohen RD. Physical activity and depression: evidence from the Alameda County Study. Am J Epidemiol. 1991;134(2):220–31. 10.1093/oxfordjournals.aje.a116074 1862805

[50] Anda RF, Williamson DF, Escobedo LG, Mast EE, Giovino GA, Remington PL. Depression and the dynamics of smoking. A national perspective. JAMA. 1990;264(12):1541–5. 2395193

[51] Luppino FS, de Wit LM, Bouvy PF, Stijnen T, Cuijpers P, Penninx BW, et al Overweight, obesity, and depression: a systematic review and meta-analysis of longitudinal studies. Arch Gen Psychiatry. 2010;67(3):220–9. 10.1001/archgenpsychiatry.2010.2 20194822

[52] Mezuk B, Eaton WW, Albrecht S, Golden SH. Depression and type 2 diabetes over the lifespan: a meta-analysis. Diabetes Care. 2008;31(12):2383–90. 10.2337/dc08-0985 19033418 PMC2584200

[53] Patten SB, Williams JV, Lavorato DH, Campbell NR, Eliasziw M, Campbell TS. Major depression as a risk factor for high blood pressure: epidemiologic evidence from a national longitudinal study. Psychosom Med. 2009;71(3):273–9. 10.1097/PSY.0b013e3181988e5f 19196807

[54] Gelenberg AJ, Hopkins HS. Assessing and treating depression in primary care medicine. Am J Med. 2007;120(2):105–8. 10.1016/j.amjmed.2006.05.059 17275446

[55] Ke CC, Lai HC, Lin CH, Hung CJ, Chen DY, Sheu WH, et al Increased Risk of Herpes Zoster in Diabetic Patients Comorbid with Coronary Artery Disease and Microvascular Disorders: A Population-Based Study in Taiwan. PloS One. 2016;11(1):e0146750 10.1371/journal.pone.0146750 26751202 PMC4709044

[56] Gilden DH, Cohrs RJ, Mahalingam R. Clinical and molecular pathogenesis of varicella virus infection. Viral Immunol. 2003;16(3):243–58. 10.1089/088282403322396073 14583142

[57] Schmader K, George LK, Burchett BM, Pieper CF. Racial and psychosocial risk factors for herpes zoster in the elderly. J Infect Dis. 1998;178 Suppl 1:S67–70. 9852978 10.1086/514254

